# Exercise during transition from compensated left ventricular hypertrophy to heart failure in aortic stenosis rats

**DOI:** 10.1111/jcmm.14025

**Published:** 2018-11-20

**Authors:** David R. A. Reyes, Mariana J. Gomes, Camila M. Rosa, Luana U. Pagan, Silmeia G. Zanati, Ricardo L. Damatto, Eder A. Rodrigues, Robson F. Carvalho, Ana A. H. Fernandes, Paula F. Martinez, Aline R. R. Lima, Marcelo D. M. Cezar, Luiz E. F. M. Carvalho, Katashi Okoshi, Marina P. Okoshi

**Affiliations:** ^1^ Botucatu Medical School Internal Medicine Department Sao Paulo State University UNESP Botucatu Brazil; ^2^ Institute of Biosciences of Botucatu Sao Paulo State University UNESP Botucatu Brazil; ^3^ School of Physical Therapy Federal University of Mato Grosso do Sul Campo Grande Brazil

**Keywords:** cardiac remodelling, echocardiogram, MAPK, oxidative stress, physical training, rat

## Abstract

We evaluated the influence of aerobic exercise on cardiac remodelling during the transition from compensated left ventricular (LV) hypertrophy to clinical heart failure in aortic stenosis (AS) rats. Eighteen weeks after AS induction, rats were assigned into sedentary (AS) and exercised (AS‐Ex) groups. Results were compared to Sham rats. Exercise was performed on treadmill for 8 weeks. Exercise improved functional capacity. Echocardiogram showed no differences between AS‐Ex and AS groups. After exercise, fractional shortening and ejection fraction were lower in AS‐Ex than Sham. Myocyte diameter and interstitial collagen fraction were higher in AS and AS‐Ex than Sham; however, myocyte diameter was higher in AS‐Ex than AS. Myocardial oxidative stress, evaluated by lipid hydroperoxide concentration, was higher in AS than Sham and was normalized by exercise. Gene expression of the NADPH oxidase subunits NOX2 and NOX4, which participate in ROS generation, did not differ between groups. Activity of the antioxidant enzyme superoxide dismutase was lower in AS and AS‐Ex than Sham and glutathione peroxidase was lower in AS‐Ex than Sham. Total and reduced myocardial glutathione, which is involved in cellular defence against oxidative stress, was lower in AS than Sham and total glutathione was higher in AS‐Ex than AS. The MAPK JNK was higher in AS‐Ex than Sham and AS groups. Phosphorylated P38 was lower in AS‐Ex than AS. Despite improving functional capacity, aerobic exercise does not change LV function in AS rats. Exercise restores myocardial glutathione, reduces oxidative stress, impairs JNK signalling and further induces myocyte hypertrophy.

## 1 INTRODUCTION

Pathological cardiac hypertrophy induced by chronic pressure overload conditions, such as aortic stenosis and arterial systemic hypertension, is often associated with impaired prognosis and increased mortality.^1^ Long‐term pressure overload is a major cause of cardiac remodelling evolving progressively from compensated left ventricular (LV) hypertrophy to diastolic and systolic dysfunction and clinical heart failure.^2^

Despite advances in the pharmacologic treatment of heart failure, morbidity and mortality levels remain elevated.^1^ Several authors and current guidelines recommend physical exercise for patients with stable heart failure to prevent and/or attenuate cardiac remodelling and skeletal muscle changes and improve functional capacity and quality of life.^3^, ^4^, ^5^, ^6^, ^7^ Physical exercise in experimental heart failure attenuated ventricular dilation, cardiac dysfunction, increased passive stiffness, myocyte hypertrophy, myocardial fibrosis, myocyte calcium handling changes and mitochondrial dysfunction.^8^, ^9^, ^10^, ^11^, ^12^, ^13^ However, as most benefits of exercise have been described in experimental models of myocardial infarction‐induced heart failure,^14^, ^15^, ^16^, ^17^ the effects of exercise during sustained LV pressure‐overload remains poorly understood. In fact, studies performed in this experimental model have returned controversial results. Aortic stenosis mice subjected to an 8‐week‐period of voluntary rotating wheel exercise showed no improvement in LV function and presented a trend towards impaired ventricular dysfunction in severe cases.^18^, ^19^ As mice perform very well in voluntary wheel running,^20^ excessive exercise may have contributed to these results. We have previously shown that aerobic exercise improves functional capacity and attenuates systolic dysfunction in rats with uncontrolled arterial hypertension^21^ or aortic stenosis.^22^

Ascending AS in rats has often been used to study chronic pressure overload. In this model, 3‐ to 4‐week‐old rats are subjected to thoracotomy and clip placement around the ascending aorta. Immediately after clip positioning, aorta diameter is preserved and stenosis progressively occurs as rats age. LV hypertrophy develops quickly, occurring within 1 month.^23^ However, ventricular dysfunction and cardiac failure develop slowly, similar to what is seen in human chronic pressure overload.^23^ This study evaluated the influence of aerobic exercise on cardiac remodelling in rats with AS performed during the period of transition from compensated LV hypertrophy to overt heart failure. An important effect of exercise is a reduction in oxidative stress^24^ associated with an attenuation in cardiac remodelling. We therefore analysed markers of systemic and myocardial oxidative stress, and myocardial antioxidant enzyme activity. The NADPH oxidase (NOX) family is a source of reactive oxygen species in various tissues including the myocardium,^25^ we therefore analysed subunits NOX2 and NOX4 gene expression. Since mitogen‐activated protein kinases (MAPK) may be involved in myocardial response to oxidative stress,^26^ we also evaluated their proteins expression in myocardium. Results for myocardial morphometric evaluation, oxidative stress analyses and protein expression from Sham and AS rats have been previously published.^27^

## 2 MATERIALS AND METHODS

### 2.1 Experimental animals and study protocol

The experimental protocol employed in this study was approved by the Animal Experimentation Ethics Committee of Botucatu Medical School, UNESP, SP, Brazil, and has previously been described in detail.^22^ Male Wistar rats (90‐100 g) were purchased from the Central Animal House, Botucatu Medical School, UNESP, and housed in a temperature controlled room at 23°C and kept on a 12‐hour light/dark cycle. Food and water were supplied ad libitum.

In short, Wistar rats (90‐100 g) were anaesthetized with a mixture of ketamine hydrochloride (50 mg/kg, i.m.) and xylazine hydrochloride (10 mg/kg, i.m.). Aortic stenosis was induced by placing a 0.6 mm stainless steel clip on the ascending aorta via a thoracic incision.^22^ Sham operated rats were used as controls. Eighteen weeks after surgery, rats were assigned to three groups: Sham (n = 23), sedentary AS (AS, n = 28) and exercised AS (AS‐Ex, n = 31) for 8 weeks. During euthanasia, we assessed the presence or absence of clinical and pathologic heart failure features. The clinical finding suggestive of heart failure was tachypnoea/laboured respiration. Pathologic assessment of heart failure included pleuropericardial effusion, left atrial thrombi, hepatic congestion, pulmonary congestion (lung weight/bodyweight ratio more than two standard deviations above Sham group mean) and right ventricular hypertrophy (right ventricle weight/body weight ratio more than 0.8 mg/g).^28^ Functional capacity was evaluated and transthoracic echocardiogram was performed before and after the exercise protocol.

### 2.2 Exercise testing

Rats underwent 10 min/day test environment adaption for 1 week before evaluations. Each animal was tested individually. The test consisted of an initial 5‐min warm up at 5 m/min on treadmill. The rats were then subjected to exercise at 8 m/min followed by increments of 3 m/min every 3 min until exhaustion. Exhaustion was determined when the animal refused to run even after electric stimulation or was unable to coordinate steps.^21^ Maximum running speed was recorded and total distance was calculated.

### 2.3 Exercise training protocol

Exercise was performed on a treadmill five times a week for 8 weeks.^21^, ^29^ There was an adaptation period, with a gradual increase in speed and exercise duration. Speed from the 1st to the 3rd week was 5, 7.5 and 10 m/min, and then remained constant until the end of the protocol. Exercise duration from the 1st to the 6th week was 10, 14, 18, 22, 26 and 30 min, and then remained constant until the end of the experiment.

### 2.4 Echocardiography

Cardiac structures and LV function were evaluated by transthoracic echocardiogram and tissue Doppler imaging using a commercially available echocardiograph (General Electric Medical Systems, Vivid S6 model, Tirat Carmel, Israel) equipped with a 5‐11.5 MHz multifrequency transducer.^30^, ^31^, ^32^, ^33^ The rats were anaesthetized with a mixture of ketamine hydrochloride (50 mg/kg) and xylazine hydrochloride (1 mg/kg) intramuscularly. A two‐dimensional parasternal short‐axis view of the left ventricle was obtained at the level of the papillary muscles. M‐mode tracings were obtained from short‐axis views of the LV at or just below the tip of the mitral‐valve leaflets, and at the level of the aortic valve and left atrium. M‐mode images of the LV were printed on a black and white thermal printer (Sony UP‐890MD, Montvale, NJ, USA) at a sweep speed of 100 mm/s. All LV structures were manually measured by the same observer (KO). Values obtained were the mean of at least five cardiac cycles on M‐mode tracings. The following structural variables were measured: left atrium diameter (LA), LV diastolic and systolic diameters (LVDD and LVSD respectively), LV diastolic (D) and systolic (S) posterior wall thickness (PWT) and septal wall thickness (SWT) and aortic diameter. LV mass (LVM) was calculated using the formula [(LVDD + DPWT + DSWT)^3^ − LVDD^3^] × 1.04. LV relative wall thickness (RWT) was calculated by the formula 2 × DPWT/LVDD. LV function was assessed by the following parameters: endocardial fractional shortening (EFS), midwall fractional shortening (MFS), ejection fraction (EF), posterior wall shortening velocity (PWSV), early and late diastolic mitral inflow velocities (E and A waves), E/A ratio, E wave deceleration time (EDT) and isovolumetric relaxation time (IVRT). A joint assessment of diastolic and systolic LV function was performed with the myocardial performance index (Tei index). The study was complemented using tissue Doppler imaging (TDI) to evaluate systolic (S′), early diastolic (E′) and late diastolic (A′) velocity of the mitral annulus (arithmetic average of the lateral and septal walls), and E/E′ ratio.

### 2.5 Collection of tissues for analysis

One day after final echocardiogram, the rats were weighed, anaesthetized with intraperitoneal sodium pentobarbital (50 mg/kg), and killed. After blood collecting, hearts were removed by thoracotomy. Atria and ventricles were dissected and weighed separately. LV was frozen in liquid nitrogen and stored at −80°C. The liver was macroscopically examined to determine the presence or absence of liver congestion. Lung weight was used to assess the degree of pulmonary congestion.^34^

### 2.6 Morphologic study

Left ventricular serial transverse 8 μm thick sections were cut in a cryostat, cooled to −20°C and stained with haematoxylin and eosin. At least 50 cardiomyocyte diameters were measured from each LV as the shortest distance between borders drawn across the nucleus.^21^ Other slides were stained with Sirius red F3BA and used to quantify interstitial collagen fraction. On average, 20 microscopic fields were analysed with a 40 X lens. Perivascular collagen was excluded from this analysis. Measurements were performed with a microscope (Leica DM LS; Nussloch, Germany) attached to a computerized imaging analysis system (Media Cybernetics, Silver Spring, MD, USA).

### 2.7 Oxidative stress evaluation

#### 2.7.1 Myocardial glutathione concentration

Reduced glutathione was determined using a kinetic method in media consisting of 100 mmol L^−1^ phosphate buffer pH 7.4 containing 5 mmol L^−1^ EDTA, 2 mmol L^−1^ 5.5′‐dithiobis‐(2‐nitrobenzoic) acid (DTNB), 0.2 mmol L^−1^ NADPH_2_ and 2 U of glutathione reductase.^35^ Total glutathione was measured in the presence of 0.1 mol L^−1^ Tris‐HCl buffer, pH 8.0 with 0.5 mmol L^−1^ EDTA, 0.6 mmol L^−1^ DTNB and 0.1 U glutathione reductase.^35^

#### 2.7.2 Malondialdehyde serum concentration

Systemic lipid peroxidation was assessed by measuring malondialdehyde (MDA) by high performance liquid chromatography (HPLC). One hundred microlitre of serum was treated with 700 μL of 1% orthophosphoric acid and vortex‐mixed for 10 seconds for protein precipitation. Then, 200 μL of thiobarbituric acid (TBA) were added. The mixture was heated to 100°C for 60 minutes and cooled to −20°C for 10 minutes. Then, 200 μL of this reaction was added to a solution containing 200 μL of NaOH: methanol (1:12). The tubes were centrifuged for 3 minutes at 13 000 *g*; 200 μL of the supernatant was taken for injection into the equipment. Analysis was performed on a Shimadzu HPLC using a C18 5 μm Gemini Phenomenex column, and an Rf‐535 fluorescence detector, which was set to Ex 525 nm and EM 551 nm. The mobile phase consisted of a 60:40 (v/v) mixture of 10 mmol potassium dihydrogen phosphate (pH 6.8): methanol. MDA was quantified by a calibration curve, which was constructed every day for analysis.

#### 2.7.3 Antioxidant enzymes activity and lipid hydroperoxide concentration

Left ventricular samples (~200 mg) were homogenized in 5 mL of cold 0.1 mol L^−1^ phosphate buffer, pH 7.0. Tissue homogenates were prepared in a motor‐driven Teflon glass Potter‐Elvehjem tissue homogenizer. The homogenate was centrifuged at 10 000 *g*, for 15 minutes at 4°C, and the supernatant was assayed for total protein, lipid hydroperoxide,^36^ and glutathione peroxidase (GSH‐Px, E.C.1.11.1.9), catalase (E.C.1.11.1.6.) and superoxide dismutase (SOD, E.C.1.15.1.1.) activities by spectrophotometry.^37^ Enzyme activities were analysed at 25°C using a microplate reader (*μ*Quant‐MQX 200) with KCjunior software for computer system control (Bio‐Tech Instruments, Winooski, VT, USA). Spectrophotometric determinations were performed in a Pharmacia Biotech spectrophotometer with temperature controlled cuvette chamber (UV/visible Ultrospec 5000 with Swift II applications software for computer system control, Cambridge, UK). All reagents were purchased from Sigma‐Aldrich (St. Louis, MO, USA).

### 2.8 Real‐time quantitative reverse transcription‐polymerase chain reaction (RT‐PCR)

Gene expression of NADPH oxidase subunits (NOX2, NOX4, p22 phox and p47 phox) and reference genes cyclophilin and glyceraldehyde‐3‐phosphate dehydrogenase (GAPDH) was analysed by RT‐PCR according to a previously described method.^38^ Total RNA was extracted from LV myocardium with TRIzol Reagent (Invitrogen Life Technologies, Carlsbad, CA, USA) and treated with DNase I (Invitrogen Life Technologies). One microgram of RNA was reverse transcribed using High Capacity cDNA Reverse Transcription Kit, according to standard methods (Applied Biosystems, Foster City, CA, USA). Aliquots of cDNA were then submitted to real‐time PCR using a customized assay containing sense and antisense primers, and Taqman (Applied Biosystems) probes specific to each gene: NOX2 (Rn00576710m1), NOX4 (Rn00585380m1), p22 phox (Rn00577357m1) and p47 phox (Rn00586945m1). Amplification and analysis were performed with Step One Plus™ Real‐Time PCR System (Applied Biosystems). Expression data were normalized to reference gene expressions: cyclophilin (Rn00690933m1) and GAPDH (Rn01775763g1). Reactions were performed in triplicate and expression levels were calculated using the CT comparative method (2^−ΔΔCT^).

### 2.9 Western blotting

Protein levels were analysed by Western blotting using specific antibodies (Santa Cruz Biotechnology, Santa Cruz, CA, USA): total JNK1/2 (sc‐137019), p‐JNK (sc‐6254), total p38‐MAPK (sc‐7972), p‐p38‐MAPK (sc‐17852), total ERK 1 (sc‐93) and p‐ERK1/2 (sc‐16982).^39^ Protein levels were normalized to GAPDH (6C5 sc‐32233). Myocardial protein was extracted using RIPA buffer (containing proteases and phosphatases inhibitors); supernatant protein content was quantified by the Bradford method. Samples were separated on a polyacrylamide gel and then transferred to a nitrocellulose membrane. After blockade, membrane was incubated with the primary antibodies. The membrane was then washed with TBS and Tween 20 and incubated with secondary peroxidase‐conjugated antibodies. Super Signal^®^ West Pico Chemiluminescent Substrate (Pierce Protein Research Products, Rockford, IL, USA) was used to detect bound antibodies.

### 2.10 Statistical analysis

Data are expressed as mean ± standard deviation or median and percentile. Comparisons between groups were performed by one‐way ANOVA and Tukey test or Kruskal‐Wallis and Dunn test. Mortality was assessed by log‐rank test (Kaplan‐Meier). Frequency of heart failure features was assessed by the Goodman test. Significance level was set at 5%.

## 3 RESULTS

Survival rate did not differ between AS and AS‐Ex groups. Heart failure features were evaluated at euthanize. The frequency of heart failure features did not differ between AS and AS‐Ex groups. Anatomical data are presented in Table 1. Both AS groups had left and right ventricular hypertrophy, and higher atria and lung weights compared to Sham. There were no differences between AS‐Ex and AS groups.

**Table 1:** Anatomical data

	Sham (n = 23)	AS (n = 19)	AS‐Ex (n = 22)
BW (g)	510 ± 50.7	482 ± 41.6	464 ± 57.5
LVW (g)	0.94 (0.88‐0.98)	1.84 (1.56‐2.09)^a^	1.48 (1.13‐1.95)^a^
LVW/BW (mg/g)	1.86 (1.69‐2.05)	3.80 (2.98‐4.33)^a^	3.19 (2.23‐5.05)^a^
RVW (g)	0.24 (0.21‐0.25)	0.46 (0.40‐0.55)^a^	0.43(0.36‐0.46)^a^
RVW/BW (mg/g)	0.48 (0.45‐0.50)	0.99 (0.83‐1.11)^a^	0.89 (0.66‐1.05)^a^
Atria weight (g)	0.10 (0.09‐0.11)	0.36 (0.21‐0.41)^a^	0.34 (0.31‐0.41)^a^
Atria/BW (mg/g)	0.20 (0.19‐0.23)	0.75 (0.66‐0.82)^a^	0.71 (0.46‐0.93)^a^
Lung weight (g)	1.80 (1.66‐1.99)	2.89 (2.11‐3.29)^a^	3.96 (2.31‐4.37)^a^
Lung/BW (mg/g)	3.80 (3.12‐4.11)	6.30 (4.29‐7.05)^a^	8.41 (4.71‐9.74)^a^

One‐way ANOVA and Tukey or Kruskal‐Wallis and Dunn test. Data are mean ± SD or median and percentile.

AS: aortic stenosis; AS‐Ex: exercised aortic stenosis; BW: body weight; LVW: left ventricle weight; RVW: right ventricle weight.

a *P* < 0.05 vs Sham.

**Figure 1:**
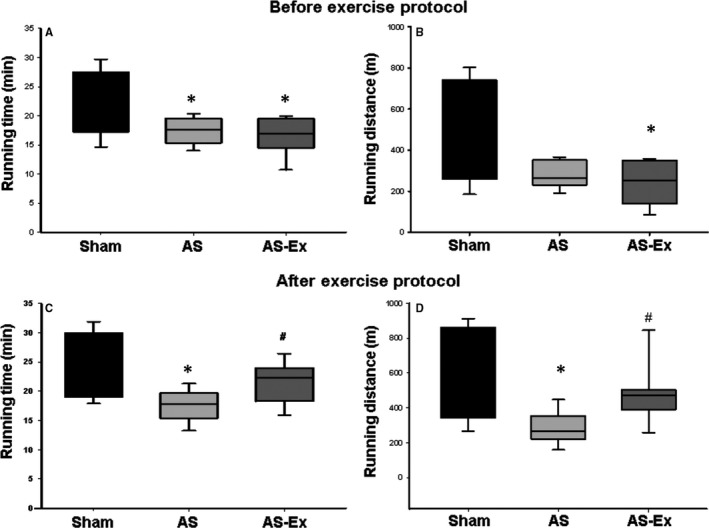
Maximal exercise test. Running time (A) and running distance (B) before exercise protocol; running time (C) and running distance (D) after exercise protocol. AS: aortic stenosis; AS‐Ex: exercised aortic stenosis. Data are median and percentile; Kruskal‐Wallis and Dunn test. *P < 0.05 vs Sham; ^#^ *P* < 0.05 vs AS

Maximal exercise test is shown in Figure 1. Before exercise, running time was lower in both AS and AS‐Ex than Sham. After exercise, running time and distance were lower in AS than Sham and AS‐Ex.

Before exercise (data not shown), AS and AS‐Ex presented left atrium dilation and concentric LV hypertrophy, characterized by increased LV wall thickness, LV mass and relative wall thickness. Both AS‐Ex and AS had LV systolic and diastolic dysfunction as posterior wall shortening velocity, isovolumetric relaxation time and E wave deceleration time were lower, and E wave and E‐to‐tissue Doppler imaging (TDI) of early diastolic velocity of mitral annulus ratio were higher than Sham. TDI S′ was lower in AS than Sham. There were no differences between AS‐Ex and AS.

Echocardiographic evaluation after the exercise protocol showed the same cardiac remodelling pattern as before exercise. Additionally, both stenosis groups presented increased LV systolic and diastolic diameters and E/A ratio, and decreased TDI S′ and A wave. Endocardial and midwall fractional shortening and ejection fraction were lower in AS‐Ex than Sham. No differences were observed between AS‐Ex and AS (Tables 2 and 3).

**Table 2:** Echocardiographic structural data after exercise protocol

	Sham (n = 23)	AS (n = 19)	AS‐Ex (n = 22)
BW (g)	504 ± 58	478 ± 50	461 ± 50^a^
LVDD (mm)	8.29 ± 0.54	9.16 ± 0.86^a^	9.11 ± 0.84^a^
LVDD/BW (mm/kg)	16.7 ± 2.04	19.3 ± 2.62^a^	20.0 ± 3.14^a^
LVSD (mm)	3.99 (3.58‐4.50)	5.04 (4.06‐5.34)^a^	5.12 (4.67‐5.99)^a^
DPWT (mm)	1.42 (1.38‐1.46)	2.05 (1.85‐2.11)^a^	2.02 (1.84‐2.17)^a^
SPWT (mm)	3.01 (2.75‐3.14)	3.48 (2.87‐3.79)^a^	3.49 (2.96‐3.74)^a^
DSWT (mm)	1.43 (1.39‐1.46)	2.11 (1.85‐2.16)^a^	2.02 (1.84‐2.17)^a^
SSWT (mm)	2.51 (2.38‐2.64)	2.82 (2.65‐3.16)^a^	3.00 (2.68‐3.25)^a^
RWT	0.34 (0.33‐0.36)	0.42 (0.40‐0.47)^a^	0.44 (0.38‐0.49)^a^
AO (mm)	4.02 (3.83‐4.16)	3.94 (3.84‐4.16)	3.97± 0.20
LA (mm)	5.29 (4.93‐5.64)	8.54 (7.72‐8.71)^a^	8.25 (7.15‐8.98)^a^
LA/AO	1.34 (1.26‐1.38)	2.17 (1.86‐2.29)^a^	2.12 (1.75‐2.25)^a^
LA/BW (mm/kg)	10.4 (9.53‐12.1)	17.4 (14.8‐19.5)^a^	17.6 (15.7‐19.5)^a^
LVM (g)	0.82 (0.76‐0.89)	1.41 (1.33‐1.96)^a^	1.61 (1.18‐1.87)^a^
LVMI (g/kg)	1.70 (1.46‐1.87)	2.97 (2.70‐3.90)^a^	3.40 (2.66‐4.16)^a^

One‐way ANOVA and Tukey or Kruskal‐Wallis and Dunn test. Data are mean ± SD or median and percentile.

AS: aortic stenosis; AS‐Ex: exercised aortic stenosis; BW: body weight; LVDD and LVSD: left ventricular (LV) diastolic and systolic diameters respectively; DPWT and SPWT: LV diastolic and systolic posterior wall thickness respectively; DSWT and SSWT: LV diastolic and systolic septal wall thickness respectively; RWT: relative wall thickness; AO: aorta diameter; LA: left atrial diameter; LVM: LV mass; LVMI: LVM index.

a *P* < 0.05 vs Sham.

**Table 3:** Echocardiographic data of left ventricular function after exercise protocol

	Sham (n = 23)	AS (n = 19)	AS‐Ex (n = 22)
HR (bpm)	291 ± 40	295 ± 41	319 ± 41
EFS (%)	51.0 (46.4‐55.3)	45.6 (38.7‐55.3)	43.8 (38.3‐48.2)^a^
MFS (%)	29.5 (27.2‐32.4)	26.8 (21.1‐33.5)	24.5 (21.6‐28.6)^a^
PWSV (mm/s)	40.6 ± 5.55	30.8 ± 6.96^a^	29.2 ± 7.15^a^
Tei index	0.44 ± 0.08	0.42 ± 0.08	0.42 ± 0.11
EF	0.88 (0.85‐0.91)	0.84 (0.77‐0.91)	0.82 (0.77‐0.86)^a^
TDI S′ (average, cm/s)	3.76 ± 0.70	2.95 ± 0.67^a^	2.92 ± 0.49^a^
Mitral E (cm/s)	79.0 (72.3‐83.5)	142 (85.0‐158)^a^	142 (98‐164)^a^
Mitral A (cm/s)	58.0 (51.5‐67.0)	25.5 (22.0‐47.0)^a^	20.0 (16.0‐58.5)^a^
E/A	1.39 (1.23‐1.52)	5.38 (1.74‐6.82)^a^	8.00 (1.49‐9.14)^a^
IVRT (ms)	26.0 (22.0‐26.0)	18.0 (15.0‐22.0)^a^	16.0 (15.0‐22.0)^a^
IVRTn	53.0 ± 7.08	42.8 ± 11.0^a^	39.6 ± 10.3^a^
EDT (ms)	46.2 ± 6.87	31.1 ± 8.67^a^	30.1 ± 8.33^a^
TDI E′ (average, cm/s)	4.51 ± 0.77	4.00 ± 1.26	4.23 ± 0.99
TDI A′ (average, cm/s)	4.49 ± 1.34	3.91 ± 1.39	4.07 ± 1.05
E/TDI E′ (average)	17.6 (14.7‐19.8)	33.5 (26.5‐41.0)^a^	34.3 (25.1‐40.4)^a^

One‐way ANOVA and Tukey or Kruskal‐Wallis and Dunn test. Data are mean ± SD or median and percentile.

AS: aortic stenosis; AS‐Ex: exercised aortic stenosis; HR: heart rate; EFS: endocardial fractional shortening; MFS: midwall fractional shortening; PWSV: posterior wall shortening velocity; Tei index: myocardial performance index; EF: ejection fraction; TDI S′: tissue Doppler imaging (TDI) of systolic velocity of the mitral annulus; E/A: ratio between early (E)‐to‐late (A) diastolic mitral inflow; IVRT: isovolumetric relaxation time; IVRTn: IVRT normalized to heart rate; EDT: E wave deceleration time; TDI E′ and A′: TDI of early (E′) and late (A′) diastolic velocity of mitral annulus.

a *P* < 0.05 vs Sham.

Myocyte diameter and interstitial collagen fraction were higher in AS and AS‐Ex than Sham. Myocyte diameter was higher in AS‐Ex than AS (Table 4).

**Table 4:** Myocardial morphometric parameters

	Sham (n = 10)	AS (n = 12)	AS‐Ex (n = 8)
Myocyte diameter (μm)	13.4 ± 1.17	15.3 ± 0.97*	16.7 ± 1.75* ^,^ #
ICF (%)	4.30 ± 1.20	9.86 ± 1.69*	8.46 ± 1.61*

One‐way ANOVA and Tukey test. Data are mean ± SD.

AS: aortic stenosis; AS‐Ex: exercised aortic stenosis; ICF: interstitial collagen fraction.

*P < 0.05 vs Sham; ^#^ *P* < 0.05 vs AS.

**Figure 2:**
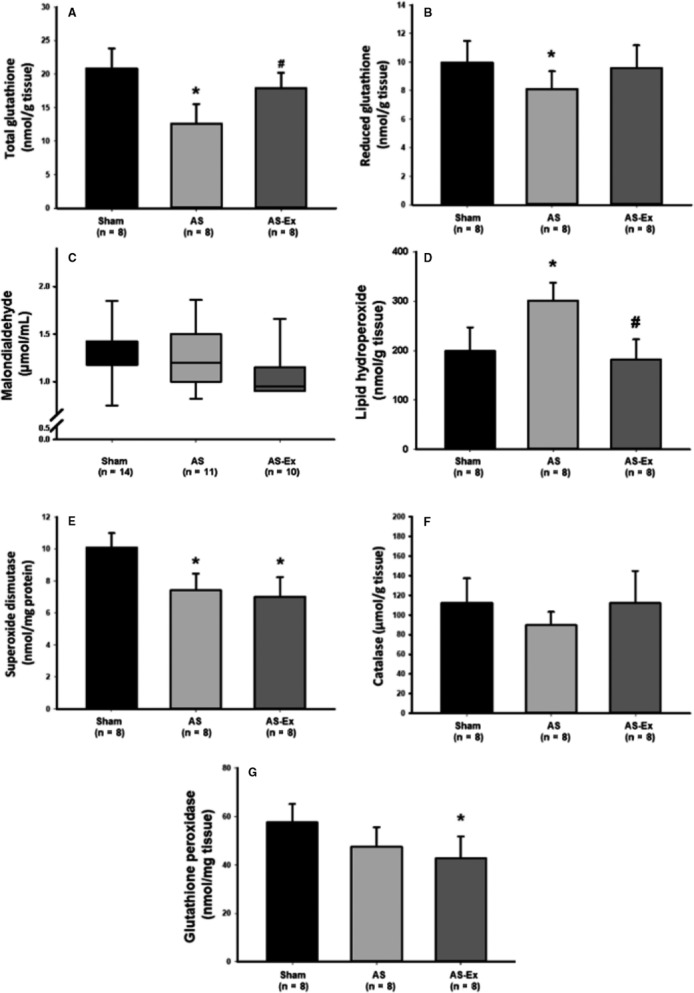
Myocardial concentration of total (A) and reduced glutathione (B); malondialdehyde serum concentration (C); lipid hydroperoxide myocardial concentration (D); and myocardial antioxidant enzyme activity: superoxide dismutase (E), catalase (F) and glutathione peroxidase (G). AS: aortic stenosis; AS‐Ex: exercised aortic stenosis; n: number of animals. Data are mean ± SD or median and percentile; ANOVA and Tukey or Kruskal‐Wallis and Dunn test; *P < 0.05 vs Sham; ^#^ *P* < 0.05 vs AS

Total and reduced myocardial concentration of glutathione was lower in AS than Sham and total glutathione concentration was higher in AS‐Ex than AS (Figure 2A and B). Malondialdehyde serum concentration did not differ between groups (Figure 2C). Lipid hydroperoxide myocardial concentration was higher in AS than Sham and AS‐Ex (Figure 2D). Myocardial antioxidant enzyme activities are shown in Figure 2 panels E, F and G. Superoxide dismutase was lower in AS and AS‐Ex than Sham and glutathione peroxidase was lower in AS‐Ex than Sham.

Myocardial gene expression of NADPH oxidase subunits did not change between groups (Table 5). Myocardial expression of MAPK proteins is shown in Table 5. Phosphorylated JNK was higher in AS‐Ex than Sham and AS groups and total JNK was higher in AS‐Ex than Sham. Phosphorylated P38 was lower in AS‐Ex than AS.

**Table 5:** Myocardial gene expression of NADPH oxidase subunits and protein expression of MAPKs

	Sham (n = 8)	AS (n = 7)	AS‐Ex (n = 8)
p22 phox	1.00 ± 0.51	1.26 ± 0.98	1.33 ± 0.71
p47 phox	1.00 ± 0.48	0.89 ± 0.61	0.74 ± 0.43
NOX 2	0.74 (0.55‐1.63)	1.64 (0.29‐4.07)	1.12 (0.50‐2.51)
NOX 4	1.01 (0.85‐1.06)	3.47 (0.19‐3.67)	1.39 (1.22‐1.82)
p‐ERK/ERK	1.00 ± 0.22	1.46 ± 0.34^a^	1.33 ± 0.20
p‐ERK/GAPDHH	1.00 ± 0.16	1.30 ± 0.85	1.74 ± 0.77
ERK/GAPDH	1.00 ± 0.21	0.98 ± 0.41	1.23 ± 0.58
p‐JNK/JNK	1.01 (0.86‐1.07)	0.78 (0.68‐1.27)	1.03 (0.94‐1.72)
p‐JNK/GAPDH	1.07 (0.95‐1.18)	1.15 (0.92‐1.26)	1.88 (1.46‐2.22)^a^^,^ b
JNK/GAPDH	1.00 ± 0.23	1.11 ± 0.19	1.48 (1.29‐1.73)^a^
p‐P38/P38	0.74 (0.50‐1.68)	1.29 (1.23‐1.81)	0.40 (0.26‐0.70)^b^
p‐P38/GAPDH	1.00 ± 0.54	2.01 ± 1.30	0.69 ± 0.41^b^
P38/GAPDH	1.05 (0.81‐1.13)	1.00 (0.70‐2.58)	1.37 (0.92‐1.92)

One‐way ANOVA and Tukey or Kruskal‐Wallis and Dunn. Data are mean ± SD or median and percentiles.

AS: aortic stenosis; AS?Ex: exercised aortic stenosis.

a *P* < 0.05 vs Sham;

b *P* < 0.05 vs AS.

## 4 DISCUSSION

In this study, we showed that aerobic exercise improves functional capacity in AS rats during transition from LV compensated hypertrophy to clinical heart failure. However, the increased physical capacity was not related to an improvement in cardiac structures or function. We also performed the first evaluation on the influence of physical exercise on myocardial oxidative stress and MAPK signalling in AS rats with heart failure.

Aortic stenosis rats have been often used to evaluate compensated LV hypertrophy and its transition to clinical heart failure. LV hypertrophy can be observed 1 month after stenosis induction.^23^ Rats then remain compensated for 20‐28 weeks when they start to present heart failure features and evolve to death in 2‐4 weeks.^40^ In this study, the exercise protocol was initiated 18 weeks after AS surgery; at this time, no rat had tachypnoea/laboured respiration. We used a low intensity exercise protocol, adapted from published studies on aged untreated spontaneously hypertensive rats.^21^, ^41^ All rats tolerated the treadmill exercise intensity of 10 m/min. The protocol was efficient as physical capacity was improved in AS‐Ex compared with AS group.

Exercise did not change heart rate or the frequency of heart failure features and survival rate. Heart rate was assessed during echocardiographic evaluation with rats under a light anaesthesia, which may have interfered with the measurement preventing heart rate reduction after exercise protocol. Before treatment, AS rats had LV concentric hypertrophy with diastolic dysfunction and mild systolic dysfunction. The same pattern of concentric hypertrophy was observed after the exercise protocol. Additionally, both AS‐Ex and AS had LV dilation and a severe degree of diastolic dysfunction. There were no differences between AS‐Ex and AS groups before and after the exercise protocol. However, AS‐Ex presented depressed LV indexes of systolic function such as endocardial and midwall fractional shortening and ejection fraction compared to the Sham group. Despite the impaired systolic function, functional capacity was increased in AS‐Ex compared with AS. Therefore, the better functional capacity in AS‐Ex was not related to an improvement in echocardiographic parameters. Studies on heart failure have suggested that exercise‐induced improvement in skeletal muscle metabolism and performance are involved in the increase of functional capacity.^22^, ^42^

Myocardial oxidative stress, evaluated by lipid hydroperoxide concentration, was higher in AS than Sham and was normalized by exercise. Exercise has been long shown to decrease oxidative stress in different experimental models.^24^, ^29^

Oxidative stress is characterized by an increase in the levels of reactive oxygen species and/or reactive nitrogen species. Both increased generation in reactive oxygen species and decreased antioxidant capacity can lead to oxidative stress.^43^ Impaired antioxidant capacity can result from low concentrations of antioxidants and decreased antioxidant enzymes activity.^43^ At physiological concentrations, reactive oxygen species play important roles in redox signalling and cell survival by modulating the activity of different enzymes such as mitogen‐activated protein kinase (MAPK), phosphatases and gene‐dependent cascades.^43^ However, high levels of reactive oxygen species cause changes or injury to DNA, proteins and lipids, and can stimulate apoptotic cell death.^24^, ^44^

Myocytes consume high quantities of oxygen during muscle cell contraction and can generate a large quantity of reactive oxygen species. Reactive oxygen species are mainly generated in mitochondria.^43^, ^44^ They can also be produced in cytosol and membranes in response to different stimuli including growth factors and inflammatory cytokines.^44^ Several enzymes including NADPH oxidase participate in ROS generation.^45^ In this study, we showed for the first time that myocardial gene expression of the NADPH oxidase subunits NOX 2 and NOX 4 is not increased in AS rats with heart failure and is not modulated by physical exercise.

Oxidative stress is neutralized by the antioxidant system, which includes endogenous and exogenous molecules. The main enzymatic defences are superoxide dismutase, catalase and glutathione peroxidase, which can be modified by exercise.^24^, ^44^ In this study, superoxide dismutase was reduced in AS and AS‐Ex compared to Sham and glutathione peroxidase was lower in AS‐Ex than Sham. Exercise preserved both total and reduced myocardial glutathione. Glutathione is an endogenous tripeptide that plays a central role in cellular defence against oxidative stress.^46^ It is synthesized and maintained at high concentrations in most cells and is the main intracellular oxidant scavenger protecting membranes, proteins and DNA from oxidative stress.^47^ In heart failure, glutathione redox status is changed and its concentration is decreased in myocardium.^48^, ^49^ Considering the restored levels of glutathione and normalization of lipid hydroperoxide, it was surprising to observe a reduced glutathione peroxidase activity in AS‐Ex compared to the Sham group. We have not identified studies evaluating physical exercise and myocardial oxidative stress in AS rats. Therefore, additional studies are necessary to clarify the interplay between exercise and oxidative stress during transition from compensated LV hypertrophy to clinical heart failure.

Phosphorylated p38 levels in AS‐Ex and AS were similar to those from the Sham group. However, phosphorylated p38 was lower in AS‐Ex than AS. Myocardial MAPK expression in rodents subjected to long‐term pressure overload and chronic exercise has been poorly addressed in literature. Miyachi et al^50^ observed that increase in p38 phosphorylation in sedentary hypertensive rats was prevented by swimming training. In accordance, it is possible that the chronic exercise has contributed to prevent an increase in myocardial phosphorylated p38 in the AS‐Ex group. As p38 activation is related to pathological hypertrophy development,^50^ its decrease in AS‐Ex group suggests a beneficial effect of exercise on the pressure‐overloaded hearts. Exercise increased myocardial protein expression of total and phosphorylated JNK. The fact that JNK activation is related to myocyte hypertrophy^51^ may explain the larger myocyte diameter in AS‐Ex than Sham and AS. However, the role of isolated JNK increase on cardiac hypertrophy is not completely obvious. JNK activity may be required for IGF‐I‐mediated cardiomyocyte growth.^52^ On the other hand, myocyte stretching stimulates JNK and inhibits IGF‐I signalling and hypertrophy.^53^ The disparate results are probably dependent on the different experimental models. Finally, myocardial fibrosis was evident in AS rats and was not modulated by physical exercise.

A large body of research spanning several decades has shown the safety and efficacy of regular physical activity in improving outcome in heart failure patients regardless of age, sex or ethnicity.^5^, ^11^, ^54^ Most clinical and experimental studies, however, have evaluated the remodelling of hearts with ischaemic cardiomyopathy.^55^, ^56^ Nonetheless, the majority of experimental studies in genetic models of systemic hypertension^21^, ^41^, ^50^, ^57^ as well as some clinical studies^58^ point towards regular exercise having a beneficial effect on cardiac remodelling. In fact, it was recently observed that exercise‐induced improvement in functional capacity is independent of heart failure aetiology.^59^ A few studies have reported negative effects of rodents subjected to long‐term exercise.^18^, ^19^, ^58^ In these studies, animals exercised by voluntary rotating wheel, which may be associated with excessive exercise.^20^ To the best of our knowledge, this is the first study to show that controlled aerobic exercise is associated with impaired cardiac remodelling in AS rats.

One important component of exercise‐induced improvement in LV function during cardiac remodelling is mediated via a reduction in peripheral impedance and cardiac afterload. However, as LV afterload is fixed in aortic constriction, this beneficial effect of exercise is not seen in aortic stenosis.^18^ The results of this study show that the beneficial effect of exercise in reducing oxidative stress and restoring glutathione was preserved in a myocardium subjected to chronic and persistent pressure overload. However, in the face of increased afterload and during the transition from compensated hypertrophy to heart failure, the further overload caused by exercise resulted in impaired MAPK pathway signalling, additional myocyte hypertrophy and a trend towards worse systolic function compared to sedentary AS rats. It is probable that the previously observed beneficial effects of exercise in chronic and persistent pressure overload^40^, ^60^ occurred early in the remodelling process and not during the transition to clinical heart failure, when rats present advanced degrees of LV hypertrophy and dysfunction, thus preventing a reverse remodelling process.

In conclusion, aerobic exercise improves functional capacity in AS rats during the transition from LV compensated hypertrophy to clinical heart failure independent of changes in LV function. Physical exercise reduces myocardial oxidative stress and glutathione peroxidase activity, and restores myocardial concentrations of total and reduced glutathione. Despite the beneficial effects on oxidative stress, exercise impairs JNK signalling and further induces myocyte hypertrophy.

## ACKNOWLEDGEMENTS

We are grateful to Jose Carlos Georgette for his technical assistance and Colin Edward Knaggs for English editing. Financial support was provided by CNPq (Proc. n. 306770/2015‐6 and 308674/2015‐4); FAPESP (Proc. n. 2014/21972‐3 e 2012/22485‐3); PROPe, UNESP; and PAEDEX/AUIP Program.

## CONFLICTS OF INTEREST

The authors confirm that there are no conflicts of interest.
